# Sensory Analysis of Full Immersion Coffee: Cold Brew Is More Floral, and Less Bitter, Sour, and Rubbery Than Hot Brew

**DOI:** 10.3390/foods11162440

**Published:** 2022-08-13

**Authors:** Mackenzie E. Batali, Lik Xian Lim, Jiexin Liang, Sara E. Yeager, Ashley N. Thompson, Juliet Han, William D. Ristenpart, Jean-Xavier Guinard

**Affiliations:** 1 Department of Food Science and Technology, University of California, Davis, 1, Shields Avenue, Davis, CA 95616, USA; 2 UC Davis Coffee Center, University of California, Davis, 1, Shields Avenue, Davis, CA 95616, USA; 3 Department of Chemical Engineering, University of California, Davis, 1, Shields Avenue, Davis, CA 95616, USA

**Keywords:** coffee, cold brew, full immersion, descriptive analysis

## Abstract

Cold brew coffee is often described as sweeter or less acidic than hot brew coffee. Such comparisons, however, are potentially confounded by two key effects: different brew temperatures necessarily change the extraction dynamics and potentially alter the resulting brew strength, and different consumption temperatures are well known to affect perceived flavor and taste. Here, we performed a systematic study of how extraction temperature affects the sensory qualities of full immersion coffee. The investigation used a 3 × 3 × 3 factorial design, with coffee from three different origins representing different post-harvest methods (washed, honey-processed, and wet-hulled), each roasted to three different levels (light, medium, and dark), and each brewed at three different temperatures (4 °C, 22 °C, and 92 °C). All coffees were brewed to equilibrium, then diluted to precisely 2% total dissolved solids (TDS) and served at the same cold temperature (4 °C). We find that four attributes exhibited statistically significant variations with brew temperature for all origins and roast levels tested, with bitter taste, sour taste, and rubber flavor all higher in hot brewed coffees, and floral flavor higher in cold brewed coffee. However, there were strong interactions with origin and roast, with several additional attributes significantly impacted by temperature for specific origins and roast levels. These results provide insight on how brew temperature can be used to modulate the flavor profile of full immersion coffee.

## 1. Introduction

Cold brew coffee is a popular beverage category, accounting for about 10% of all coffee sales in the United States [1]. However, the precise definition of cold brew coffee is a source of some confusion. Traditionally, coffee is brewed hot for a fast extraction time, either using a drip brew, a pressurized system like espresso, or a full immersion brew such as French press. In instances where consumers prefer a cold coffee beverage, hot brewed coffee can be quickly prepared then refrigerated or poured over ice to instantly chill it. In contrast, “cold brew” coffee is most typically a full immersion method that involves steeping coffee grounds in chilled or room temperature water for up to 24 h to sufficiently extract the soluble compounds into the beverage. There is widespread belief that cold brew coffee is sweeter and less acidic (both chemically and sensorially) than chilled hot brew coffee, driving its recent popularity [2].

Hot or cold, consumers highly value the sensory quality of coffee [3], so understanding how brewing can maximize desirable flavors and minimize undesirable flavors is vital information for coffee professionals. There is a wide variety of potential sensory attributes in coffee, as tabulated in the World Coffee Research Sensory Lexicon [4] and displayed in the Coffee Taster’s Flavor Wheel [5]. The great diversity of flavors can be attributed to a large variety of compounds in green and roasted coffee that have been characterized for their sensory qualities; comprising a mix of carbohydrates, acids, lipids, proteins, antioxidants, and volatile aroma compounds [6]. A variety of factors can influence these flavors, including the coffee origin [7,8], processing [9], degree of roasting [10], and final brew methods [10,11,12,13,14,15,16].

With regard to brew method, brew temperature is believed by coffee professionals to influence sensory quality, but quantitative data is limited. For hot brew, the Coffee Brewing Handbook states that coffee needs to be brewed at 92 °C to 96 °C for adequate extraction [17]. Recent research contradicts that claim, however; detailed descriptive analysis [13] and consumer preference tests [18] indicated that at a fixed strength (total dissolved solids, or TDS) and extraction (percent extraction, or PE), brew temperature ranges from 87 °C to 93 °C yielded almost indistinguishable sensory profiles and consumer acceptance [13,18]. In other words, it did not matter what temperature was used to brew the coffee provided that other parameters like the grind size and water flow rate were varied so that the coffee was brewed to the same TDS and PE. That research only focused on a narrow range of temperatures between 87 to 93 °C, raising the question: Does brewing temperature over a wider range of temperatures affect the sensory profile at fixed TDS and E regardless of how quickly the strength is obtained?

Early research by Pangborn and collaborators touched on this question. They showed that at a fixed TDS, and in a temperature range of 65–100 °C, coffee brewed at 80 °C and above was assessed as more bitter and sour [19]. More recent work comparing hot brewed coffee to traditional cold brewing temperatures (approximately 2 to 25 °C) has generally indicated that lower temperature brews are sweeter and/or less acidic. Cordoba et al. evaluated the effect of brewing temperature by comparing cold immersion, cold drip, and hot brewed French press, and found that the French press and cold immersion had lower TDS and higher sweetness when consumed at 19 °C, and cold immersion had a lower titratable acidity [20]. Consumer preference evaluation by Heo et al. found consumers preferred ready-to-drink cold brews, characterized by dark chocolate, hazelnut, and sweet aromatics, whereas fresh brewed cold brew was perceived as higher in citrus fruit flavors [21]. Angeloni et al. compared cold brew and cold drip at room temperature (22 °C) and refrigerator temperature (5 °C) to hot brewed (95 °C) French press coffee and found that the cold brew coffees had a higher intensity of caramelized and sweet attributes and were also more sour than the hot brewed French press coffee [22].

This prior work, however, typically did not compare cold versus hot brews both at fixed TDS and PE, as well as at fixed serving temperature. It is well established that consumption temperature will change flavor perception of coffee [23,24], so an otherwise identical coffee will be perceived differently if served hot versus cold. Likewise, a substantial amount of research shows that brewing the same coffee to different TDS and PE values will vastly change the sensory profile [14,16,18,25]. Less strong, lower TDS coffee has repeatedly been shown to be sweeter than higher TDS coffees [14,16,25], while sourness and bitterness increased with TDS [13,14,16]. Titratable acidity was shown to correlate positively with TDS independent of brew temperature in hot brewed coffee, at least over the range 87 to 93 °C [15]. What remains unclear is how brew temperature impacts the sensory profile of coffee over a wider range of temperatures, especially those customarily used for cold brew coffee, when TDS, PE, and serving temperature are removed as confounding variables.

Therefore, the main goal of this study was to characterize how the sensory attributes of coffee change with brewing temperatures at fridge temperature (4 °C), room temperature (22 °C), and hot temperature (92 °C), all served at a fixed TDS and fixed consumption temperature (4 °C, typical refrigeration temperature) to remove those confounding effects. Additionally, since coffee origin and roast level are large contributors to the sensory properties of coffee, we sought to identify any interactions between brew temperature and origin or roast level, to determine if certain coffee types respond differently for cold brewing or hot brewing. We hypothesized that even at a fixed TDS and consumption temperature, brew temperature would still play a role in the final sensory properties of coffee, due to differences in taste and flavor compound solubility in cold water. Corollary hypotheses were that some sensory attributes would be roast or origin dependent, considering different initial concentrations of sensory-active compounds in the roasted coffee, and that unique flavor attributes would be highlighted or suppressed depending on temperature.

## 2. Materials and Methods

### 2.1. Summary of Experimental Design

To systematically investigate what affects the flavor of brewed coffee, we used a classic factorial experimental design [26] to examine a wide range of parameters. Green coffees from three different origins were each roasted to three different levels, and then each was brewed at three temperatures, resulting in 3 × 3 × 3 = 27 coffees. Each sample was then assessed by physical measurements (TDS, titratable acidity [TA], and pH) and by sensory descriptive analysis. Each of the 27 samples was prepared in triplicate to accommodate three tasting and analytical replicates (*n* = 81).

### 2.2. Coffees

For this experiment, three green *Coffea arabica* samples were used: El Salvador Cerro Las Ranas Honey varietals Bourbon, Pacamara, Sarchimor, Pacas, Catuai, and Caturra (ELS), an Ethiopia Guji Washed organic of indigenous heirloom varietals (ETH), and a Sumatra Fair-Trade Organic Takengon varietals Catimor, Tim Tim, and Abyssinia (SUM). These specific coffees were chosen both due to geographic differences and also because of the vast differences in their postharvest processing: the ELS coffee was honey-processed (or semi-dry processed), where some fruit and pulp of the coffee cherry remain during the drying process; the ETH was a fully washed (or wet-processed) coffee, where the mucilage is removed with water, and the SUM was ‘wet-hulled’ where both the fruit and the parchment of the coffee cherry are removed prior to drying [27].

For the roast factor of the experiment, each of the three green coffees were roasted to three different levels representing a “light”, “medium”, or “dark” roast. Roasting was done in a single day per origin on a Probatino P-5 (Probat-Werkevon Gimborn Maschinenfabrik GmbH, Emmerich am Rhein, Germany). Roast color was measured with an Agtron, with light roasts having an Agtron Gourmet score of 58, medium roasts an average score of 48, and dark roasts an average score of 38 (Agtron E2OCP-II Coffee Analyzer, Agtron Inc., Reno, NV, USA). Roasted beans were allowed to de-gas for a week before storage in vacuum-sealed bags placed in a freezer maintained at −20 °C to maintain a consistent sensory quality [28].

### 2.3. Brewing Procedure

One day prior to roasting, bags of coffee were removed from the freezer and allowed to equilibrate to room temperature over the course of 24 h. All coffees were ground to a grind size of 4 on a Mahlkönig Guatemala Lab Grinder (Mahlkönig USA, Durham, NC, USA), corresponding to a median particle size of 972 ± 19 microns (cf. Supplemental Figure S1 in ref [29]).

**Figure 1:**
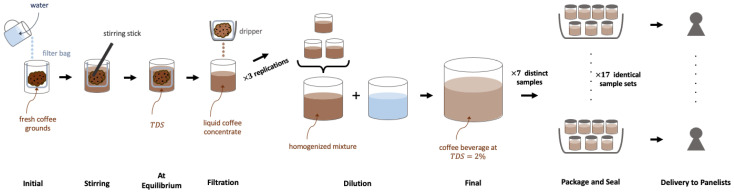
A schematic of the coffee brewing process and packaging for a remote COVID-19 descriptive analysis.

The brewing and serving procedures can be visualized in Figure 1. Coffees were brewed using Toddy Cupping Kits (Toddy LLC., Loveland, CO, USA) with Toddy paper filters. All coffees were brewed with a brew ratio $R_{brew}=$ 5, with 100 g of coffee grounds to 500 g of Nestlé Pure Life Purified Water with a TDS of 53 ppm (Nestlé, Issy les Moulineaux, Paris, France). Three Toddy Cupping kits per brewing condition were used, totaling 300 g coffee and 1500 g water per sample. For the 92 °C brews, water was heated to 92 °C in a Bonavita 1.7-L Variable Temperature Electric Kettle (Bonavita World, Woodinville, WA, USA). For both the fridge brews and room temperature brews, room temperature (22 °C) water was initially added to the coffee grounds. Immediately after adding the room temperature water to the coffee grounds, the 4 °C brews were placed in the fridge; this protocol reflects standard industry practice. For each temperature, the coffee was stirred immediately after adding water by hand at approximately 2 rotations per second for several seconds to ensure adequate mixing.

After mixing, the coffees were left undisturbed at their respective holding conditions, with the goal of letting them brew completely to extraction equilibrium [29]. The fridge brews were stored for 24 to 36 h during brewing; the 22 °C brews sat at room temperature for 12 to 14 h; and the initially 92 °C coffees sat at room temperature for 1 to 2 h. The fridge and room temperatures were measured to vary by no more than ±1 °C, while the hot brew was allowed to cool without additional heating to reproduce typical conditions used by consumers and in the coffee industry.

After the coffee had reached equilibrium (with TDS values ranging from about 3.8 to 4.6%, see Table 1), the filter and grounds were removed and placed on top of the brewing vessel in a metal filter to allow all the liquid to drain from the grounds through the filter paper. Once all liquid was drained, coffees from the three separate brewing vessels were combined and the TDS measured. The combined brew was then diluted to precisely 2% TDS. Samples for individual panelists were then separated into individual 4oz Ball mason jars (Ball Corporation, Westminster, CO, USA) and stored in the fridge overnight (at 4 °C) to ensure that the serving temperature was the same for all brews.

### 2.4. Physical and Chemical Measures

All coffees were brewed to equilibrium TDS, but that equilibrium value varied depending on the coffee origin and roast level. Pre-dilution physical measures are shown in Table 1. TDS was measured using a VST LAB Coffee III digital refractometer (VST inc, Boston, MA, USA) with two drops of coffee centrifuged at 6000 rpm for 30 s in a Thermo Scientific MySPIN 6 mini centrifuge (Thermo Fisher Scientific, Waltham, MA, USA), at room temperature. The refractometer was calibrated in accordance with procedures outlined in [29]. PE at equilibrium was calculated from the measured TDS as Equation (1):

(1) $$\mathrm{PE}=\frac{\mathrm{TDS}}{1-\mathrm{TDS}}\times R_{\mathrm{brew}}.$$

This version of the calculation for PE is appropriate at the relatively strong brew ratios used here because it avoids the confounding influence of retained liquid in the spent grounds (cf. Equations (17) and (24) in [29]).

Chilled 250 mL samples of coffee were used to measure pH and titratable acidity the day after brewing. The pH measurements were taken of the sample while stirred, using a Mettler Toledo S220 SevenCompact™ Benchtop pH/ISE Meter (Mettler Toledo, Greifensee, Switzerland). Titratable acidity was then measured by adding 0.1 M NaOH (Sigma Aldrich, St. Louis, MO, USA) dropwise from a burette while the 50 mL sample of coffee was stirred, until reaching a pH of 8.24 ± 0.06 Titratable acidity is expressed here in mL NaOH/50 mL coffee. The pH meter was calibrated using acidic (VWR chemicals, pH 4.00 ± 0.01, color coded red, VWR international, Radnor, PA, USA), neutral (VWR chemicals, pH 7.00 ± 0.01, color coded yellow, VWR international, Radnor, PA, USA), and basic (VWR chemicals, pH 10.00 ± 0.01, color coded blue, VWR international, Radnor, PA, USA) calibration standards before every set of six measurements.

### 2.5. Descriptive Analysis Protocol

Training and evaluation protocols were modified from a traditional descriptive analysis to adhere to COVID-19 social distancing guidelines, as approved under UC Davis revised IRB protocol 1082569-2. Ballot development and training took place over video conference in Fall of 2020. Panelists received sets of 8 coffees either by delivery or picking up at our facility the morning of the first training session of the week, with the instructions to keep samples refrigerated and sealed until the tasting session. During the first two weeks of tasting, panelists were split into two groups of 10 for video conference sessions where they tasted 4 coffees at a time and underwent free term generation as inspired by the Coffee Taster’s Flavor Wheel [5]. After a set of 27 terms was selected (Table 2), panelists participated in further discussion and training to agree on and become familiar with reference standards for all attributes included in the final ballot. During the last week of training, panelists received references delivered to their houses with the coffee to work with them directly while tasting coffee. All references and standards are listed in Table 2. Finally, panelists underwent two practice tasting sessions at home to confirm panel alignment before starting the formal descriptive analysis.

During the actual evaluation, 17 remaining panelists received 7 samples twice a week. Each evaluation day, they were emailed a code to log into RedJade (RedJade, Redwood City, CA, USA) where they were prompted with a three-digit blinding code to taste each sample. All samples were tasted in triplicate in a Williams Latin Square block design. Panelists were instructed to taste coffee one at a time, in one sitting, in an area of their home free from noise or smells. The participants rated each attribute on an unstructured line scale from 0–100, with anchors at 10 and 90. Fourteen of the 17 panelists completed all sessions, and four were dropped due to poor performance (i.e., inconsistency between replicates) for 10 panelists left in the analysis.

### 2.6. Data Analysis

Descriptive analysis results were exported from Red Jade, converting positions on the 15-cm line scale into scores from 0 to 100 for each attribute. R version 4.0.2 (22 June 2020)—“Taking Off Again” (R Core Team, 2019) was used for a three-factor analysis of variance with two-way interactions among judges, replicates, and samples to determine which attributes were significant, at α = 0.05. The F values were adjusted from the original ANOVA through a pseudo-mixed ANOVA to calculate new F ratios and determine which attributes were significant despite a judge effect. Attributes with too high a judge by sample interaction (i.e., not significant in the pseudo-mixed ANOVA) were dropped from further analysis. Data was then analyzed using a 5-factor analysis of variance, with roast, origin, temperature, judge, and replicate as factors. Coffee means for all factors determined to be significant and not excluded for judge effect by pseudo-mixed ANOVA were then compared using Fisher’s Least Significant Difference (LSD) through the Agricolae package in R. Principal component analysis biplots derived from the matrix of mean significant sensory attribute ratings across coffees were produced in R using the FactoMineR and ggplot2 packages (Figure S1).

## 3. Results

### 3.1. Physicochemical Measures

All coffees were brewed to extraction equilibrium (i.e., where TDS no longer increases), but the strength at equilibrium varied by both temperature and roast, as shown in Table 1. Overall, hot brewed coffees extracted more material than the colder brews, with an average TDS of 4.27% and equilibrium PE of 22.28% (averaged over all roast levels and origins). In contrast, the room temperature brews had an average TDS of 3.98% and equilibrium PE of 20.74%, while the cold brews had the lowest values with a TDS of 3.76% and PE of 19.55%. In other words, the hot water extracted on average approximately 13% more soluble material than the coldest water. Roast level also affected the extraction slightly; the light roast coffees systematically had higher equilibrium TDS and PE than the darker roasts, albeit by not as large a difference as between brew temperatures. Origin had little effect on the equilibrium extraction. We emphasize, however, that to account for these differences in equilibrium extraction all coffees were diluted to precisely 2% TDS before consumption.

**Figure 2:**
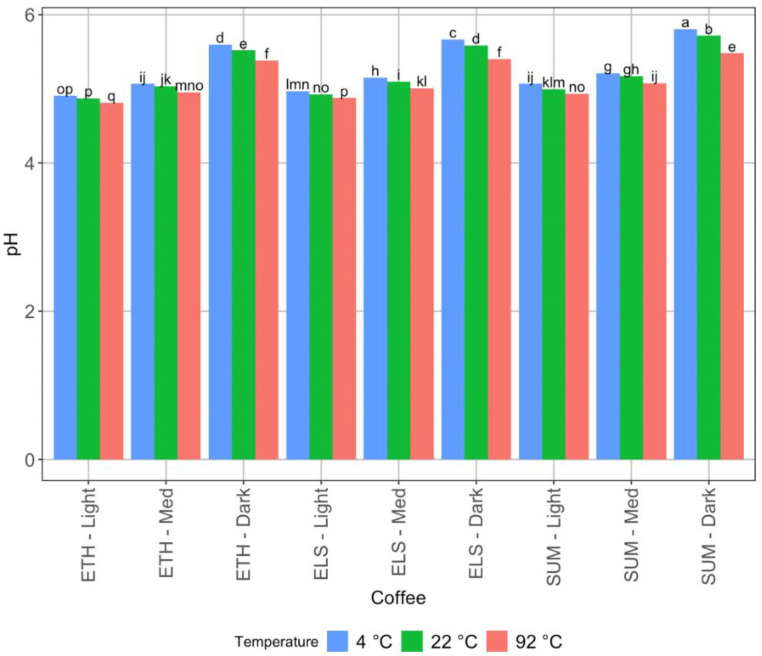
The mean pH value of all brews, with LSD letter codes to indicate significant difference. Codes refer to origin (Eth, ELS, and SUM as Ethiopia, El Salvador, and Sumatra respectively), roast (L, M, or D as light, medium, or dark respectively), and brew temperature (4 °C, 22 °C, and 92 °C).

**Figure 3:**
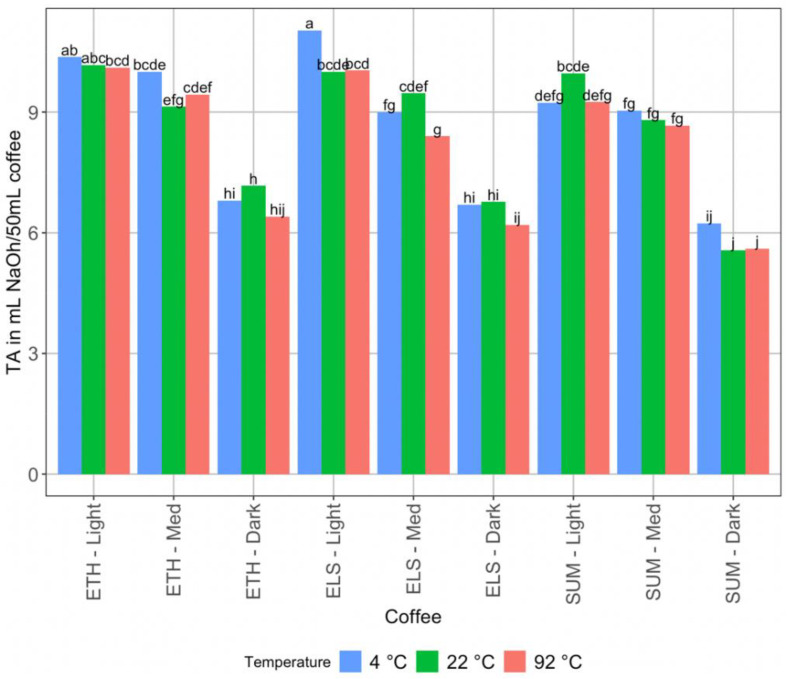
The mean titratable acidity of all brews, with LSD letter codes to indicate significant difference.

The acidity of the brews also varied significantly. Consistently, the higher temperature samples (92 °C) had a lower pH than the 22 °C and 4 °C samples (Figure 2). In most cases, the 22 °C coffee was also significantly lower in pH and therefore more acidic than the 4 °C coffee, but in some cases (Ethiopia light roast, El Salvador light roast, and Sumatra medium roast), the 22 °C and 4 °C coffees were not significantly different. Titratable acidity (Figure 3) only varied significantly with roast level, not with brew temperature—for most coffees, there were no significant differences in titratable acidity by brewing temperature. However, for El Salvador (honey processed) light roast, the 4 °C brew had significantly higher acidity than the 22 °C brew or the 92 °C brew. For El Salvador (honey processed medium roast), the 22 °C brew was more acidic than the 92 °C brew, though not significantly more than the 4 °C brew, and the 4 °C brew was not significantly higher than the 92 °C brew.

### 3.2. Sensory Measures: Roast and Origin

Overall, in the 5-way ANOVA, and not surprisingly, roast was the largest driver of difference among the coffees, with 20 attributes showing a significant difference by roast level (Table 3). In general, the lighter roasts were more floral, fruity, and sour, whereas the darker roasts were more burnt, bitter, and roasted (Figure S1).

Origin was the second largest driver of difference of the three experimental factors, with the 5-way ANOVA revealing 13 attributes that were significantly different by origin. The honey-processed El Salvador and washed Ethiopia coffees, for example, were generally more sour and fruitier than the wet-hulled Sumatra coffees, which were more vegetative and nuttier. Many of the differences by origin, however, were also dependent on the roast and brew temperature, as discussed in more detail below.

### 3.3. Sensory Measures: Temperature

**Figure 4:**
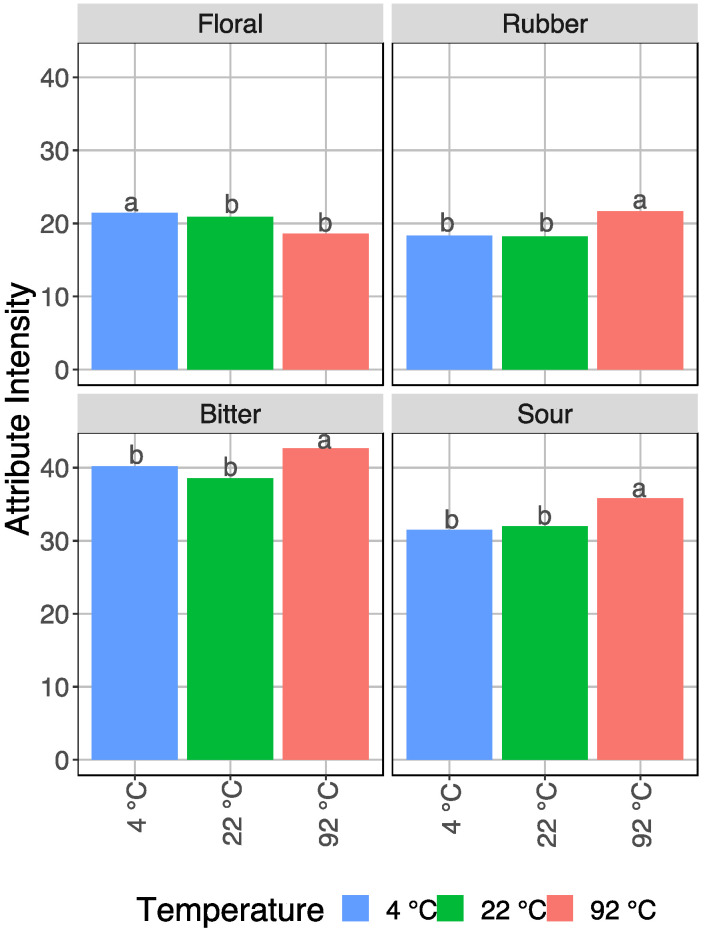
Sensory attributes with significant differences by temperature across all roasts and origins with LSD letter codes indicating significant difference.

Overall, in the 5-way ANOVA (Table 3), across all origins and roasts, four attributes showed significant differences across all roasts and origins by brewing temperature alone: floral flavor, rubber flavor, bitter taste, and sour taste. Figure 4 shows the mean intensity for each brew temperature for each of these attributes across all roasts and origins. Floral flavor was significantly higher (*p* < 0.05%) in the 4 °C brew than in the 22 °C brew or the 92 °C brew (differences indicated by LSD letter codes). Rubber flavor, bitter taste, and sour taste follow the opposite trend, where the intensity is significantly higher in hot brewed coffee, while the 4 °C and 22 °C samples were not significantly different in those attributes.

**Figure 5:**
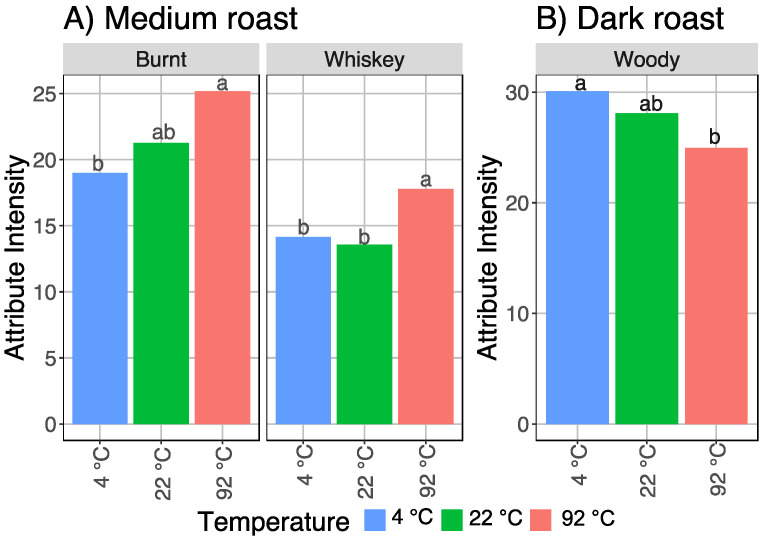
The attributes significantly different by brew temperature for individual roast levels, with LSD letter codes; (**A**) medium roast; (**B**) dark roast.

The data was also examined by roast to determine if certain attributes only changed with temperature within certain roast levels (Figure 5). The light roast coffee did not show any additional differences aside from the 4 attributes that were different across all roasts. Medium roast coffee also exhibited an increase in burnt flavor with brew temperature, with 92 °C brew significantly higher than 4 °C brew. Likewise, whiskey flavor in the medium roast was significantly higher in the 92 °C brew than in the 4 °C and 22 °C brews. For the dark roast coffee, woody flavor was significantly higher in the refrigerator brewed coffee, while the room temperature coffee was not significantly different from either refrigerator or hot.

**Figure 6:**
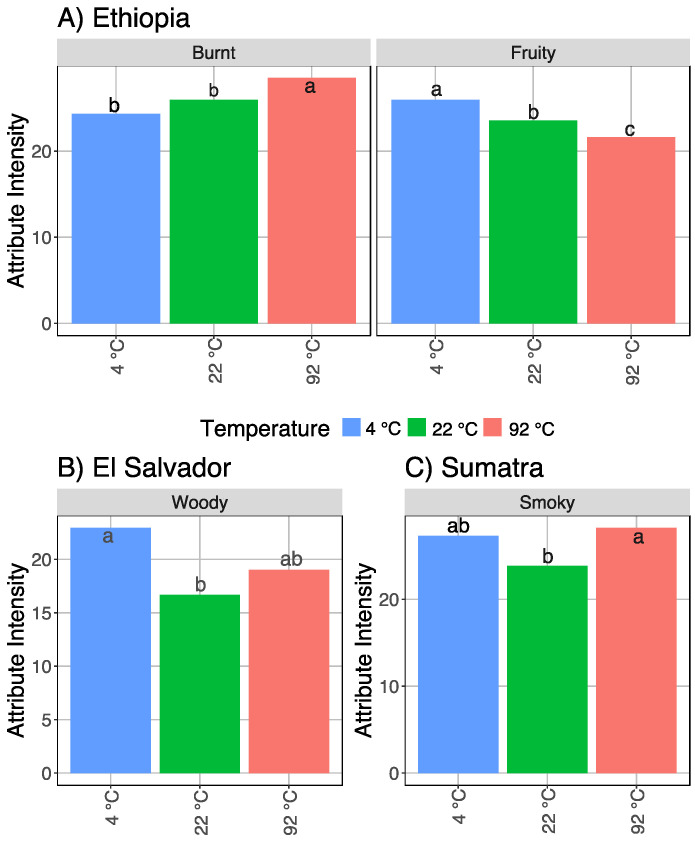
The attributes significantly different by brew temperature for individual origins, with LSD letter codes; (**A**) Ethiopia; (**B**) EI Salvador; (**C**) Sumatra.

In addition to the four attributes that varied consistently by brew temperature, each origin showed unique temperature differences (Figure 6). The Ethiopia coffee across all roast levels showed a significantly higher burnt flavor in the 92 °C coffee than the 22 °C coffee or the 4 °C coffee. Conversely, fruity flavor was significantly higher in the 4 °C coffee than the 22 °C or the 92 °C coffee. The El Salvador coffee only had one additional attribute, woody flavor, that was significant across all roasts, which followed a slightly different trend: the 4 °C coffee had the highest woody intensity, the 22 °C coffee was significantly lower, and the 92 °C coffee was intermediate. The Sumatra coffee also had one significant attribute across all roasts, smoky flavor, which also followed a pattern similar but reversed to the El Salvador’s woody flavor. In the Sumatra coffee, the 92 °C coffee had the highest smoky flavor, with the 22 °C coffee significantly lower and the 4 °C coffee in the middle.

### 3.4. Principal Component Analyses

**Figure 7:**
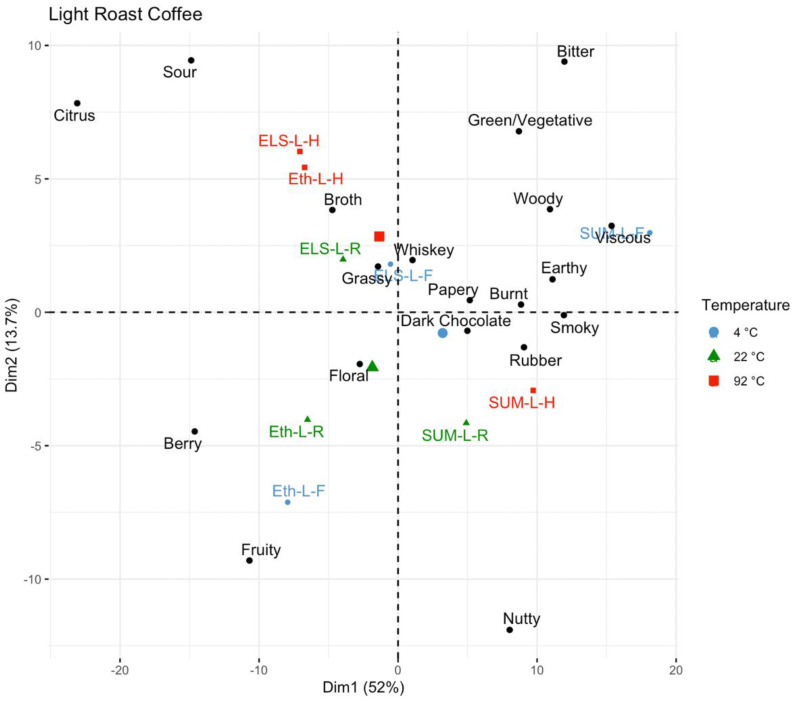
A principal component analysis of the light roast coffees, color-coded by brew temperature.

Temperature is seen as a driver of difference in the multivariate analysis, with the PCA for the light roast coffee (Figure 7) showing separation by temperature and varying by origin. The SUM-L-4C sample is in the upper left associated with woody, rubber, smoky, and burnt attributes, whereas the ETH-L-4C is lower right with floral and fruity, and ELS-L-4C is in the upper right associated with grassy and broth. In general, the higher temperatures tend to be closer to the center of the plot, indicating that for all three of these origins at light roast, brewing at 4 °C highlights unique sensory attributes that become less prominent at 22 °C and 92 °C brewing temperatures. SUM-L-92C is a slight exception, farther in the lower left where it is associated with nutty flavor.

**Figure 8:**
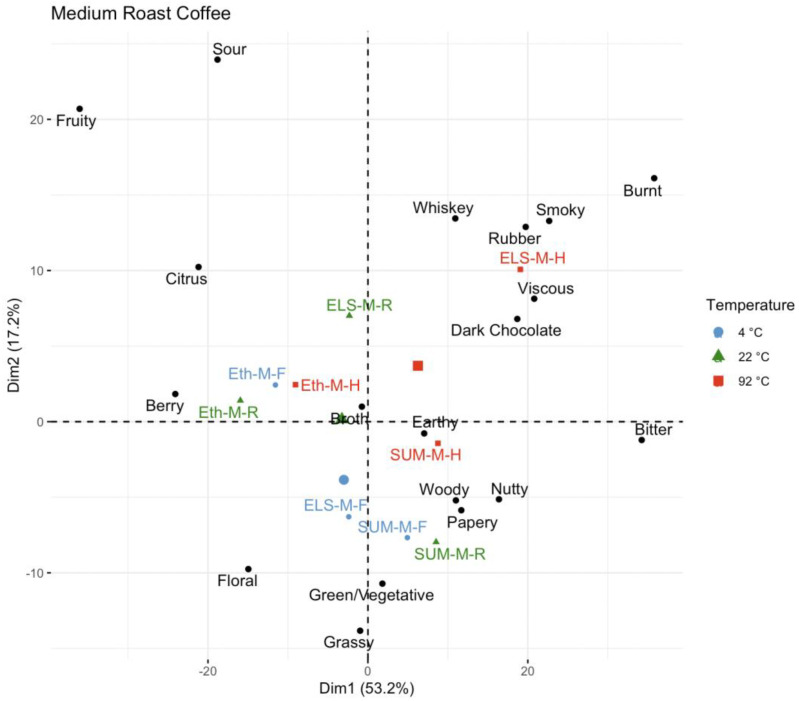
A principal component analysis of medium roast coffees, color-coded by brew temperature.

Principal component analysis of the medium roast coffee (Figure 8) again highlights how the temperature dependence of the coffee varies by origin. The Ethiopian (washed) medium roast coffees vary little with temperature, separated only slightly on dimension 2, but all three temperatures on the left side of dimension 1 associated with fruity, citrus, berry, and floral. Similarly, Sumatra (wet hulled) coffees do not have a large separation, with SUM-M-4C and SUM-M-22C very closely clustered in the bottom center of the product space associated with grassy, earthy, and papery flavors, while the SUM-M-92C coffee is higher and to the right in the product space closer to woody, dark chocolate, and green/vegetative attributes. The El Salvador (honey) coffee has the most separation among the medium roast samples, with the 4 °C and 22 °C samples centered on the product space, while the 92 °C sample is much farther in the upper right associated with smoky, burnt, rubber, viscous, bitter, and whiskey attributes.

**Figure 9:**
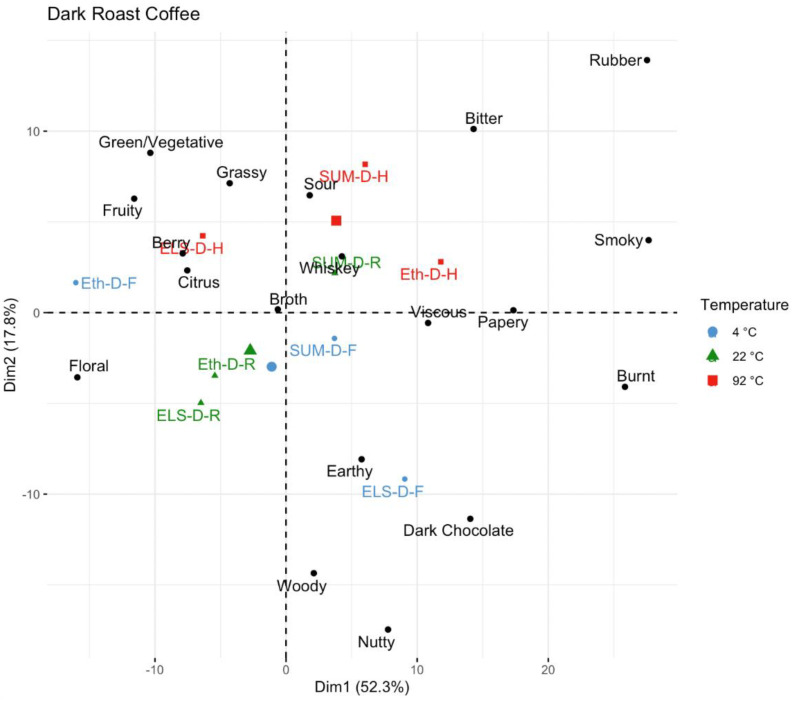
A principal component analysis of dark roast coffees, color-coded by brew temperature.

Finally, for the dark roast coffee (Figure 9), origin appears to be a large driver of difference, but within origins for Ethiopia (washed) and El Salvador (honey) there is a large amount of variation by temperature as well. The ETH-D-4C coffee is separated in both directions from the ETH-D-92C coffee, with the 4 °C coffee in the lower left associated with floral flavor, and the 92 °C coffee in the upper right associated with bitter, rubber, and smoky attributes. The El Salvador (honey processed) is separated on the opposite diagonal, with the fridge brewed ELS-D-4C in the lower right quadrant associated with dark chocolate, whiskey, and nutty attributes, and the ELS-D-92C in the upper left quadrant associated with citrus, fruity, sour, and broth flavors. The ELS-D-22C is also uniquely separated, in the lower left nearer to the ETH-D-4C sample associated with floral flavor. For the dark roast Sumatra (wet hulled), Figure 6 does not show nearly as much separation as ELS and ETH, with all three temperature levels right of center on dimension 1, associated with burnt, papery, dark chocolate, whiskey, and nutty attributes.

## 4. Discussion

It is not surprising, based on previous literature and industry knowledge, that roast was the biggest driver of difference among coffees in this study. Previous sensory literature has shown many significant differences for the same coffee roasted to different levels [7,8,16,20,30]. It is well understood that chemical reactions in roasting contribute to the flavor profile of coffee, and these reactions continue as the roast goes on to change precursors in green coffee to new flavors in roasted coffee of varying levels. Additionally, we had three different origins in this study, and origin was the next most substantial driver of difference, which likewise is unsurprising given that different regions have different terrain, soil microbiology, and weather patterns which will all contribute to flavor [31,32,33,34]. Furthermore, the different origins also underwent different postharvest processing methods (washed, honey, and wet-hulled), which have been well documented to contribute to the flavor profile in coffee [9,35,36,37,38,39,40]. Ultimately, our results confirmed existing and known differences between roasts and origins.

With regard to brew temperature, our work established that four sensory attributes changed significantly regardless of roast or origin. Three attributes were higher in intensity as the brew temperature was increased—rubber flavor, bitter taste, and sour taste—and one attribute, floral, increased as the brew temperature was decreased. Although we did not perform any chemical analysis in this study, existing literature provides some possible mechanistic hypotheses to explain these observed sensory trends.

**Rubber flavor:** Perception of rubber flavor in coffee has been shown to be the strongest driver of penalties to consumer liking [25]. One possible explanation for rubber flavor might be differences in the concentrations of 2,4-dimethyl-1,3-thiazole, (ethyldisulfanyl)ethane, or styrene, all compounds shown to correlate with rubber flavor [41]. While extraction of these compounds individually may not be heavily dependent on temperature, coffee is a complex matrix of volatile and nonvolatile compounds, and it is possible there is some interaction.

**Sour taste:** With regards to sour taste, most organic acids, particularly those abundant in coffee that contribute to sourness, are readily soluble in water of all temperatures, and the solubility regardless of temperature is reflected in the minimal differences in titratable acidity by temperature. However, some bitter and sour components have been shown to have an additive effect, such as increasing concentrations of caffeine increasing perception of the sourness of citric acid, so the increase in bitterness might likewise increase the perception of sour tasting compounds, even if they were not increased notably in concentration [42].

**Bitter taste:** Differences in solubility at different temperatures likely influence the extraction of bitter compounds and consequent perception of bitter taste. There are a large number of identified bitter compounds in coffee including caffeine, chlorogenic acid lactones, and trigonelline [43,44]. These all have temperature dependent solubilities; caffeine, for example, increases in solubility as temperature increases. Chlorogenic acid lactones are also major contributors to bitterness, and there is no literature reporting specific solubility values of these compounds, so further study on whether or not there are differing levels of these lactones or other bitterants would be valuable. Furthermore, the additive impact with sourness could also have impacted bitter perception depending on any differences in acid extraction.

**Floral flavor:** The final attribute, floral flavor, potentially comes from compounds such as phenylacetaldehyde, 4-(4′-hydroxyphenyl)-2-butanone, (E)-β-damascenone, linalool, geraniol, or other volatile compounds [45] that have been identified in coffee. We speculate that the higher perception of floral flavor in cold brew coffee was due to two factors. These compounds are all volatile, and the increase in temperature will increase volatility, which may have caused more aroma loss in the hot brew before the brew is completed and chilled. Secondly, the higher intensity of bitter and sour tastes might have decreased the perception of the floral flavors in the hot brewed coffee via a masking effect, even if the volatile compounds are there in equivalent amounts.

One possible factor that influenced our results involves the color of the brews. A concurrent experimental study [46] that we performed examined the colorimetric properties of the same coffees brewed for this study. The main finding was that cold brewed coffee tends to be redder and hot brewed coffee tends to be blacker. Color can play a role in sensory perception [47], particularly considering these coffees were not consumed under controlled lighting conditions due to the COVID protocol. Therefore, the slightly redder appearance may have contributed to the cold brew coffee being perceived as less bitter.

Another factor to consider in these temperature differences is percent extraction. While the samples were all diluted to the same TDS, coffees were brewed to an equilibrium TDS where further extraction ceased. We emphasize that this equilibrium TDS and corresponding PE differed for each temperature or roast level, with dark roast cold brews averaging the lowest PE values, and light roast hot brews the highest (cf. Table 2). In hot drip brew coffees, bitterness and rubber flavor increase with both TDS and PE [13,16]. Sour flavor, on the other hand, consistently has a negative relationship with PE, with low PE coffees perceived as sourer [13,15,16]. We emphasize this trend is the opposite of the trend observed in the current study, where our cold brew coffees had a lower PE than the hot brews and were also less sour. Likewise, drip brew coffee with high PE is also perceived as slightly more floral than coffee with low PE [25], which again is the opposite of the trend seen here with colder brews having lower PE and a higher floral flavor. The results indicate that a rule of thumb appropriate for hot drip brews—that lower PE means higher sourness—does not apply to full immersion brews over a wider range of temperatures.

Beyond the four attributes that varied with temperature regardless of roast and origin, this study also confirmed that different coffee types vary strongly in how brew temperature impacts the final sensory properties. Some coffees, like medium roast coffees for all three origins, and the Ethiopia (washed) coffee for all three roast levels, all exhibited a significant increase in burnt flavor in hot brewed coffee. This is potentially a negative sensory attribute [18,25], indicating that some consumers might prefer these coffees brewed cold. In other coffees, we saw a higher perception of attributes like woody flavor in dark roast and the El Salvador (honey) 4 °C brews, and fruity flavor in the Ethiopia (washed) coffee at 4 °C. Our results indicate a potential trend of cold brew better preserving or showcasing unique aromas in coffee. However, one confounding factor may be due to the length of the hot brew, leaving a full immersion to equilibrium required up to an hour of extraction time. Typically, an immersion coffee brew like in coffee cupping will be tasted around 5 min after the brew begins [48]. Aroma compounds are volatile, and the long time left at a higher temperature prior to chilling might have resulted in loss of compounds responsible for attributes like woody, burnt, or floral flavors and aromas. However, if the differences between hot and cold brew were in part due to aroma loss before chilling in the hot brew in this experimental design, the issue may not apply to coffee brewed directly over ice as is another common practice.

Finally, the pH and titratable acidity results in comparison to the sourness left some questions unanswered. The pH results followed conventional wisdom that cold brew is less acidic than hot brew, but the titratable acidity, usually correlated with sourness [15], did not vary significantly different between cold and hot brewed samples. Perceived sourness then went up with brew temperature as well. We speculate that brew temperature impacts the concentrations of different kinds of acids, as there are many different acids in coffee with varying sensory properties [49]. Further work with detailed chemical analysis of the acids present is needed to elucidate the reason behind these discrepancies.

## 5. Conclusions

This research showed that at a fixed TDS and consumption temperature, brew temperature across the cold to hot range had a significant impact on the sensory properties of coffee. The results presented here corroborate the general belief that cold brew is less sour, per our descriptive analysis, but not necessarily less acidic chemically. Different roast levels and origins also exhibited different changes in sensory attributes with temperature, indicating that depending on individual or consumer preference, cold or hot brew might be better for different coffee types. There were far fewer differences between room (22 °C) and refrigerator (4 °C) temperature brews than between either of those temperatures and the hot (92 °C) brews. Our results improve the limited existing body of cold brew academic literature and provide rigorous data to inform industry training about cold brew, allowing better understanding of how to brew coffee with different desired sensory properties.

## Figures and Tables

**Table 1:** Means and standard deviations for all physical and chemical measurements for each sample in the sensory study.

Origin	Roast	Brew Temperature	Equilibrium TDS (%)	Equilibrium PE (%)	Undiluted Brew Mass (g)	Diluted TDS (%)	Diluted Brew Mass (g)	Diluted pH	Diluted Titratable Acidity (mL NaOH)
ELS	Light	92 °C	4.30 ± 0.02	22.47 ± 01	922.57 ± 5.14	1.97 ± 0.05	1988.73 ± 45.67	4.88 ± 0.02	10.03 ± 0.45
		22 °C	4.01 ± 0.04	20.98 ± 0.2	934.10 ± 16.85	1.97 ± 0.06	1916.83 ± 79.55	4.93 ± 0.02	10.00 ± 0.36
		4 °C	3.78 ± 0.04	19.64 ± 0.2	886.70 ± 31.87	2.00 ± 0.03	1670.20 ± 73.52	4.97 ± 0.02	11.03 ± 0.45
	Medium	92 °C	4.20 ± 0.06	21.92 ± 0.3	910.50 ± 33.05	2.04 ± 0.04	1252.73 ± 45.92	5.01 ± 0.01	8.40 ± 0.87
		22 °C	3.90 ± 0.05	20.29 ± 0.3	924.67 ± 22.43	1.97 ± 0.06	1819.87 ± 40.58	5.10 ± 0.01	9.47 ± 0.68
		4 °C	3.81 ± 0.15	19.80 ± 0.75	914.27 ± 38.05	1.91 ± 0.06	1569.50 ± 183.58	5.15 ± 0.02	9.00 ± 0.79
	Dark	92 °C	4.13 ± 0.07	21.54 ± 0.35	844.23 ± 13.62	2.00 ± 0.06	1762.83 ± 46.86	5.40 ± 0.02	6.20 ± 0.20
		22 °C	3.74 ± 0.04	19.43 ± 0.2	859.53 ± 13.92	1.99 ± 0.04	1682.00 ± 220.19	5.59 ± 0.01	6.75 ± 0.78
		4 °C	3.50 ± 0.04	18.13 ± 0.2	803.87 ± 22.04	1.97 ± 0.03	1375.17 ± 55.30	5.66 ± 0.03	6.70 ± 0.61
ETH	Light	92 °C	4.45 ± 0.18	23.29 ± 0.9	978.27 ± 10.90	1.99 ± 0.05	1438.43 ± 23.16	4.81 ± 0.02	10.10 ± 0.00
		22 °C	4.21 ± 0.05	21.98 ± 0.25	974.63 ± 29.84	2.00 ± 0.07	2061.47 ± 79.13	4.87 ± 0.01	10.17 ± 0.91
		4 °C	4.06 ± 0.11	21.16 ± 0.55	976.67 ± 11.21	2.00 ± 0.08	1976.87 ± 79.10	4.91 ± 0.03	10.37 ± 0.57
	Medium	92 °C	4.31 ± 0.02	22.52 ± 0.1	957.97 ± 15.63	2.02 ± 0.06	1355.56 ± 46.43	4.95 ± 0.01	9.43 ± 0.50
		22 °C	4.08 ± 0.08	21.27 ± 0.4	970.47 ± 19.05	1.99 ± 0.06	1916.55 ± 5.44	5.04 ± 0.01	9.13 ± 0.71
		4 °C	3.89 ± 0.13	20.24 ± 0.65	940.57 ± 56.19	1.97 ± 0.01	1829.00 ± 133.72	5.07 ± 0.02	10.00 ± 0.35
	Dark	92 °C	4.06 ± 0.16	21.16 ± 0.8	909.77 ± 8.45	2.02 ± 0.03	1235.03 ± 114.19	5.38 ± 0.06	6.40 ± 0.66
		22 °C	3.73 ± 0.08	19.37 ± 0.4	897.20 ± 28.35	1.96 ± 0.04	1642.27 ± 75.32	5.52 ± 0.02	7.17 ± 0.32
		4 °C	3.46 ± 0.04	17.92 ± 0.2	842.63 ± 44.74	1.99 ± 0.01	1436.37 ± 81.69	5.60 ± 0.02	6.80 ± 0.30
SUM	Light	92 °C	4.42 ± 0.04	23.12 ± 0.3	951.43 ± 18.96	1.97 ± 0.01	2069.60 ± 46.44	4.94 ± 0.02	9.25 ± 0.21
		22 °C	4.23 ± 0.09	22.08 ± 0.45	965.83 ± 17.85	2.01 ± 0.05	2027.80 ± 90.72	5.00 ± 0.01	9.97 ± 0.91
		4 °C	4.00 ± 0.11	20.83 ± 0.55	916.27 ± 36.11	1.97 ± 0.03	1816.33 ± 129.27	5.07 ± 0.10	9.23 ± 0.59
	Medium	92 °C	4.30 ± 0.03	22.47 ± 0.15	925.17 ± 10.80	1.97 ± 0.04	2014.93 ± 77.48	5.08 ± 0.02	8.67 ± 0.38
		22 °C	4.11 ± 0.13	21.43 ± 0.65	938.80 ± 20.66	1.98 ± 0.06	1274.60 ± 26.91	5.17 ± 0.02	8.80 ± 0.40
		4 °C	3.78 ± 0.03	19.64 ± 0.15	892.07 ± 26.31	1.99 ± 0.01	1126.76 ± 55.05	5.21 ± 0.04	9.03 ± 0.15
	Dark	92 °C	4.23 ± 0.06	22.08 ± 0.3	878.37 ± 31.17	1.98 ± 0.04	1846.73 ± 80.59	5.48 ± 0.02	5.60 ± 0.36
		22 °C	3.83 ± 0.05	19.91 ± 0.25	867.60 ± 4.96	1.95 ± 0.00	1680.97 ± 65.24	5.72 ± 0.01	5.57 ± 0.40
		4 °C	3.58 ± 0.05	18.56 ± 0.25	816.40 ± 46.90	1.99 ± 0.02	1445.57 ± 81.41	5.81 ± 0.03	6.23 ± 0.25

**Table 2:** Sensory attributes chosen for the study and corresponding reference standards prepared for the panelists.

Attribute	Description/Reference
Bitter	Basic taste of bitter (0.1% caffeine solution in water)
Sour	Basic taste of sour (1.25% citric acid solution in water)
Sweet	Basic taste of sweet (2% sugar solution in water)
Astringent	Mouth-drying, puckering feeling (2% alum solution in water)
Viscous	Thick mouthfeel (verbal descriptor)
Black Tea	Brewed Lipton black tea
Floral	Dry Celestial Seasonings chamomile lavender tea
Berry	Fresh mixed berries
Citrus	Fresh sliced lemon and orange
Fruity	Signature Select mixed fruit (White Grape Juice From Concentrate, Pineapple, Peaches, Pears, Cherry)
Brown Sugar	Signature Select dark brown sugar
Smoky	Wright’s liquid smoke in water
Burnt	French Roast coffee beans
Roast/Grain	Roasted barley and cereal, crushed
Nutty	Justin’s almond butter
Earthy	Damp soil
Papery	Damp cardboard
Fermented	Guinness beer
Dark Chocolate	Ghirardelli 99% dark chocolate
Green/Vegetative	Fresh sliced green pepper and cucumber
Broth	Better than Bouillon (vegetable)
Rubber	Bike tube, rubber bands
Whiskey	Jim Beam Whiskey
Molasses	Grandma’s Molasses
Brown Spice	Ground cinnamon, nutmeg, cloves
Woody	Fresh cut wood, wood chips
Grassy	Fresh cut grass

**Table 3:** The F values for the experimental factors in the five-way ANOVAs, bolded to indicate significance and adequate panel agreement calculated by pseudomixed ANOVA.

	Origin	Roast	Temperature
Floral	**3.59**	**24.17**	**4.75**
Brown Sugar	1.25	0.007	2.16
Dark Chocolate	1.06	**40.73**	0.33
Black Tea	0.21	9.72	0.08
Berry	**11.04**	**39.06**	1.36
Fruity	**22.11**	**78.26**	1.02
Citrus	**12.87**	**142.17**	1.71
Rubber	**4.69**	**146.9**	**5.76**
Papery	**3.51**	**37.84**	0.31
Nutty	2.97	**9.95**	1.13
Roast/Grain	1.08	11.11	2.10
Molasses	0.19	3.66	2.23
Green/Vegetative	0.94	**24.49**	1.98
Whiskey	0.18	**22.37**	3.03
Fermented	1.15	13.90	1.73
Broth	0.61	7.92	1.04
Grassy	0.04	**15.20**	1.61
Burnt	4.15	243.71	2.69
Smoky	**4.20**	**118.71**	2.48
Woody	1.41	**48.89**	2.5
Earthy	2.12	**74.53**	0.58
Brown Spice	0.81	8.42	3.46
Bitter	**7.20**	**178.17**	**6.06**
Sour	**11.13**	**242.64**	**8.61**
Sweet	1.59	5.57	2.27
Viscous	**5.36**	**182.77**	0.87
Astringent	0.65	0.51	0.83

## Data Availability

The data presented in this study are available on request from the corresponding author.

## Supplementary Materials

The following supporting information can be downloaded at: https://www.mdpi.com/article/10.3390/foods11162440/s1, Figure S1: PCA biplot of all roast levels, temperatures, and origins.
