# Supplementary figures and images for: Sensory Analysis of Full Immersion Coffee: Cold Brew Is More Floral, and Less Bitter, Sour, and Rubbery Than Hot Brew
Source: Foods. 2022 Aug 13;11(16):2440. doi: 10.3390/foods11162440 (PMC9407127; doi:10.3390/foods11162440)

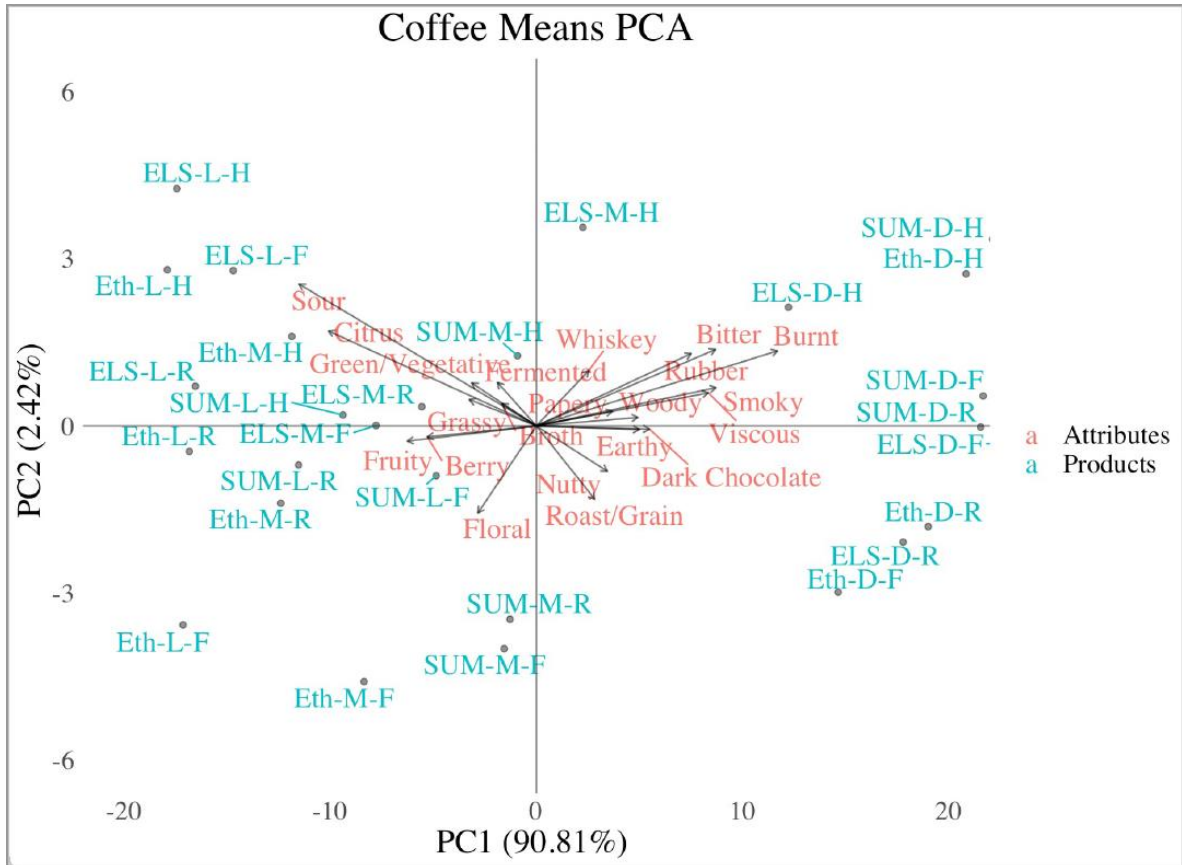

**Figure S1.** PCA biplot of all roast levels, temperatures, and origins.

Supplement: Supplementary file 1 [file foods-11-02440-s001.zip › foods-1818856-supplementary.pdf]
